# Michigan Market Referral Coordination Initiative: a Regional Market Approach to VA Specialty Care

**DOI:** 10.1007/s11606-023-08112-8

**Published:** 2023-03-20

**Authors:** Alice Cusick, Julie Gronek, Mary Gorman, Mark S. Hausman, Richard J. Schildhouse

**Affiliations:** 1 VA Ann Arbor Healthcare System, Medicine Service (111), 2215 Fuller Road, Ann Arbor, MI 48197 USA; 2 Department of Internal Medicine, University of Michigan Medical School, Ann Arbor, MI USA; 3 Battle Creek VA Medical Center, Battle Creek, MI USA; 4 Department of Anesthesiology, University of Michigan Medical School, Ann Arbor, MI USA

**Keywords:** Veterans, Access, Care coordination

## Abstract

**Background:**

The Maintaining Internal Systems and Integrated Outside Networks (MISSION) Act of 2018 was created in response to reports of prolonged wait times for veterans accessing health care within the Veterans Affairs (VA) system. In Michigan, the MISSION Act Community Care Program led to an increased number of veterans receiving specialty care outside the VA system, in part due to the complicated process of coordinating specialty care within the VA system. From 2018 to 2020, the percentage of veterans referred to the VA Ann Arbor Healthcare System (AA) for specialty care from its two referring facilities, Battle Creek VA Medical Center (BC) and Saginaw VA Healthcare System (SAG), decreased from 54.4 to 27%.

**Objective:**

Improve the number of Michigan veterans choosing VA specialty care.

**Intervention:**

In 2021, three VA facilities in Michigan (AA, BC, and SAG) created a market-level referral system named the Michigan Market Referral Initiative (MMRCI). This unique approach used a centralized nurse-driven team to manage specialty referrals, working directly with the veteran to explore both VA and community care (CC) options.

**Main Measures:**

Referrals triaged and acceptance rates for VA care were tracked. The localized Standard Episode of Care model was used to estimate cost savings. Post-intervention AA patient wait times were compared to local CC wait times.

**Key Results:**

In the 14 months after implementation of the MMRCI, the rate of veteran retention increased by 32.4%. The estimated dollars retained within the VA by MMRCI efforts was $24,105,251 as of 7/1/2022. Post-intervention AA wait times were superior to community care except in 3 specialties.

**Conclusions:**

This multifacility effort is an example of a highly coordinated, veteran-centered collaboration that has led to successful retention of veterans within the VA system with resultant large-scale cost avoidance and comparable clinic wait times. Focusing on central care coordination and veteran engagement in the referral process are keys to its success, along with leveraging existing referral patterns between nearby VA facilities. This model could be extrapolated to other VA markets throughout the country where similar relationships exist.

## INTRODUCTION

The Veterans Affairs (VA) medical system is the largest integrated healthcare system in the USA, caring for over 9 million veterans among 18 regions called Veterans Integrated Services Networks (VISNs). Each VISN consists of multiple healthcare facilities of varying complexity localized to a geographic area. Historically, when a specialty service was unavailable within a veteran’s local facility, the veteran was referred to another nearby VA facility, either within or outside the VISN.

In 2014, the Office of the Inspector General published a report on significant problems with scheduling primary and specialty care at the VA medical center in Phoenix, AZ.^1^ Prolonged wait times for care were also noted nationally throughout the VA medical system, and later that year Congress passed the Veterans Access, Choice, and Accountability Act of 2014, creating the VA Choice Program. This program allowed veterans access to private sector services utilizing their VA benefit if certain distance or wait time criteria were met. The Maintaining Internal Systems and Integrated Outside Networks (MISSION) Act of 2018 expanded VA Choice by creating the “Community Care Program” (CCP) to coordinate referrals for care outside the VA system.

The CCP has not fully solved issues with veterans’ access to medical and surgical specialty care. Clinical limitations include sharing key medical information and results, and issues with obtaining medications, as well as the lack of a comprehensive program overseeing quality of care.^2,3^ Administrative hurdles cited by VA directors are multiple and include slow reimbursement to community providers, a cumbersome secondary authorization, the scheduling program, and previous negative experiences with the VA Choice program as challenges for community care (CC) providers.^4^ Unfortunately, these experiences with difficult care coordination are common consequences of delivering care across multiple healthcare systems, both within and outside the VA system.

Financial vulnerability for veterans utilizing the CCP became a noteworthy problem when they received bills for CC not covered by the VA. Given the protracted and complex appeals process, many veterans have been left with unexpected debt.^4^ In addition, the cost of CC has been rising dramatically. According to the Congressional Budget Office, the VA spent 7.9 billion dollars on CC in fiscal year (FY) 2014 and 17.6 billion dollars in FY 2021, an increase of 122% during a time when the number of enrolled veterans only increased from 9.1 to 9.2 million.^5^

Also, similar to private sector patients, the lack of primary care and specialty community providers in rural areas makes accessing CC particularly difficult for rural veterans.^6,7^ After implementation of the MISSION Act of 2018, wait times for primary and specialty care even in urban areas did not substantially differ between the VA and private sector.^8^ While wait times vary based on location, in the majority of VISNs veterans can consistently access care in the VA more quickly than in community-based clinics.^9^

It became clear that access to CC care alone did not satisfy all veterans’ medical needs, and many veterans reported a preference to stay within the VA system even if they were eligible for CC.^10^ However, veterans were often not made aware of the option to stay within the VA system for specialty care, even if it could be provided virtually or through a neighboring VA facility. In Michigan, the veterans’ lack of awareness was due in part to the cumbersome specialty care referral system. In a state where the majority of VA specialty clinics are in Ann Arbor and Detroit, most veterans in outlying VA clinics are eligible for CC under the MISSION Act. After the MISSION Act was implemented in 2019, primary care providers (PCPs) found referring veterans to CC to be simpler and less time-consuming

In order to increase the number of veterans choosing to receive care within the VA, in 2019 the VA implemented the “Referral Coordination Initiative” (RCI). In this initiative, responsibility for referral management was shifted away from overburdened referring providers.^10^ Each VA healthcare system has approached the RCI process differently. Herein we describe the VA Ann Arbor Healthcare System (AA) approach.

## METHODS

### Setting

The AA main campus is in Ann Arbor, MI, and serves as a tertiary referral center for veterans in the lower peninsula of Michigan, northern Ohio, and northern Indiana. Historically, 40% of the AA facility workload came from providing specialty care services for the Saginaw VA Healthcare System (SAG) in Saginaw, MI, and the Battle Creek VA Medical Center (BC) in Battle Creek, MI. SAG covers a large area of 35 rural Michigan counties, while BC covers a mix of urban and rural veterans throughout 22 counties. While they do offer limited specialty services, both SAG and BC focus on foundational services such as primary care and mental health. The majority of veterans served by BC and SAG live greater than 1 h from AA when traveling by car. A 60-min average drive time to specialty care is one of the criteria used to qualify a veteran for CC under the MISSION Act of 2018; therefore, BC and SAG veterans routinely qualify for CC.

The referral process to AA for specialty services utilizes an interfacility consult (IFC) which historically has been seen as complex, time-consuming, and burdensome for SAG and BC PCPs, leading to much frustration. By contrast, after implementation of the MISSION Act of 2018, the referral process to CC was much simpler, and thus AA saw a sharp decline in referrals for specialty services from BC and SAG (42% and 41.5% respectively). In parallel, BC and SAG increased overall CC referrals for specialty services during the same period.

### Intervention

In 2021, AA piloted an RCI process by engaging with BC and SAG to create a centrally coordinated referral system. This unique approach of involving three separate VA health systems was renamed the Michigan Market Referral Coordination Initiative (MMRCI). A Michigan Market Referral Coordination Team (MMRCT) in the AA Office of CC was established, utilizing existing staff to coordinate referrals for specialty services with both the PCP and the veteran.

The pilot MMRCI had three goals: (1) simplify the specialty care referral process for PCPs, (2) engage directly with the veteran to ensure the veteran is aware of specialty care options within the VA, and (3) increase the number of Michigan veterans choosing to utilize VA specialty care.

While the Computerized Patient Record System (CPRS) is the national VA electronic health record, each VA facility has local control of CPRS, leading to differences in ordering menus and consult templates. Therefore, prework between facilities was needed prior to MMRCI implementation. This work included alignment of CPRS consult titles and templates, reviewing and updating service agreements, and establishing specialty specific order sets for use by MMRCT members. Extensive education to front line staff and MMRCI huddles between sending and receiving facilities were also completed prior to implementation. The timeline for deployment of these components within the sending facility was approximately 6 weeks prior to “go-live” for each specialty service.

The MMRCI utilized existing workforce members for this pilot project. No separate facility funding was provided under the RCI directive.

**Figure 1:**
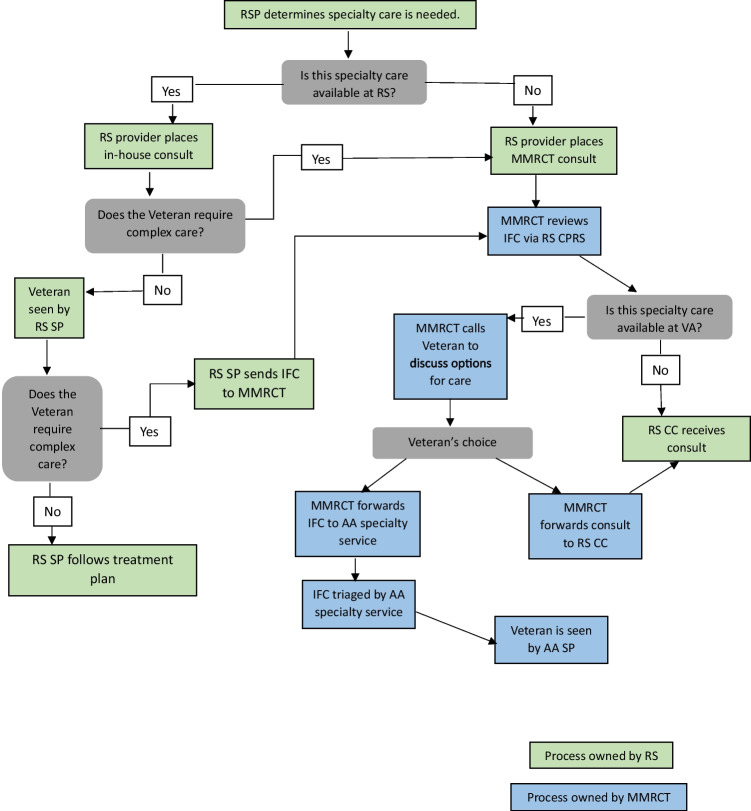
Michigan Market Referral Coordination process map. RSP, referring site provider; RS CC, referring site community care; MMRCT, Michigan Market Referral Coordination team; CC, community care; IFC, interfacility consult; CPRS, Computerized Patient Record System; AA SP, Ann Arbor Specialty Provider.

Figure 1 outlines the MMRCI workflow process. All IFCs for specialty services are reviewed by the MMRCT, and if the requested specialty service is available in AA the MMRCT contacts the veteran to offer a referral to AA or to a community provider based on their preference. The MMRCT is well-versed in the virtual care services as well as travel and lodging assistance programs available at AA. This allows real-time adjudication of the distance barrier if that is cited as a concern by the veteran.

Virtual care options in Michigan are multiple and include VA Video Connect (at-home video visits directly with veterans), Clinical Video Teleconference (video visits with the veteran from a distant VA clinic), and E-consults (where a specialist asynchronously reviews a veteran’s chart and provides advanced recommendations for care to the referring provider).

As a care coordination team, the nurses in MMRCT have access to the CPRS systems of BC, SAG, and AA, and can order any laboratory or imaging studies required for the IFC, along with initiating outside records requests. By engaging directly with the veteran, MMRCT ensures the veteran is fully aware of their options within the VA. Thus, MMRCT serves the dual role of both referral coordinator and care navigator. For veterans who opt to maintain specialty care within the VA, individual AA specialty clinics triage the IFC per their standard consult procedures. If a veteran chooses to use their VA benefit to obtain specialty care in the community, any provider preferences they have are documented and their referral is directed to their home VA facility’s CCP for scheduling.

On May 24, 2021, the MMRCI process was implemented in BC for Otolaryngology, Hematology, Oncology, Vascular Surgery, and Nephrology. The MMRCI for these same five specialties was implemented at SAG on December 9, 2021.

Other specialties were subsequently integrated into MMRCI for BC, including Neurosurgery (6/29/21), General Surgery and Rheumatology (7/28/21), Plastic Surgery (9/20/21), and Pulmonology (11/10/21).

### Measures

Pyramid Analytics (Pyramid Analytics Inc., Amsterdam) was utilized for data collection regarding consults. Within the consult database of Pyramid Analytics, the dimensions of Receiving Site, File Entry Date, and Consult Service Name were searched, producing measures of the number of IFCs for each specialty sent from BC and SAG and accepted to AA or directed back to the referring CCP. This information was tracked monthly for each sending site throughout the entire evaluation period from May of VA FY 2021 to June of VA FY 2022. Subsequently, retention was calculated for each specialty service by dividing the number of IFCs who opted to stay within VA over the total number of IFCs.

Estimated costs were calculated by specialty service using the average Standard Episode of Care (SEOC) cost, which is the amount VA reimburses to community providers for bundled services associated with each specialty referral. This information was retrieved from the Advanced Medical Cost Management System for the most recently completed fiscal quarter, and the average SEOC cost was multiplied by the number of IFCs to AA for the specialty by referring site, as they are location specific.

AA new patient wait times were checked on 8/28/2022, 8 months after the last AA clinic implemented the MMRCI process. This date was chosen to mark when the initiative was at steady state. Wait times were assessed via the Corporate Data Warehouse, a database that delivers visibility and availability across departments in VA. AA wait times were compared to known community wait times utilizing VISN 20 Care Management Tool that was developed to measure new patient wait times from file entry date to scheduled appointment in the community.

We did not collect wait times prior to MMRCI implementation. As wait times are dynamic and complex, we were unable to obtain historical wait times for comparison.

## RESULTS

Table 1 illustrates the BC and SAG specialty referral retention rates by AA for the 3 years prior to MMRCI implementation and in the 14 months afterwards. In FY 2018, a total of 54.4% of referrals from BC and SAG remained in the VA system. After the MISSION Act implementation in FY 2019, retention rates decreased to 35.2% in FY 2019 and to 27% in FY 2020.

**Table 1:** Specialty Care Ann Arbor Referral Retention Before and After MMRCI Implementation

Fiscal year	AA retention from BC (%)	AA retention from SAG (%)	Total (%)
2018	73.9	45.9	54.4
2019^*^	39.6	33.2	35.2
2020	30.4	24.6	27.0
Post-MMRCI	31.2	35.0	32.2

^*^MISSION Act of 2018 implemented

*BC*, Battle Creek; *SAG*, Saginaw; *CC*, community care; *AA*, Ann Arbor

After the MMRCI implementation, 8925 consults from BC were processed with 2283 (31.2%) veterans opting to receive care within the VA system. Otolaryngology, Nephrology, and Neurosurgery received the most consults at 1492, 1429, and 1176, respectively. The highest rates for VA specialty retention were in Hematology (58%) and Rheumatology (43%).

While Plastic Surgery was involved in the MMRCI, their data are not presented in the results. There is no CC Plastic Surgery consult in the BAC and SAG systems so the retention rate could not be calculated.

In the 7-month period after the MMRCI implementation at SAG a total of 2876 consults were processed with 1017 veterans (35%) opting to maintain care within the VA system. Otolaryngology, Hematology, and Nephrology received the most consults at 744, 744, and 685, respectively. The highest rates for VA specialty retention were Otolaryngology (40%), Hematology (39%), and Rheumatology (39%).

Table 2 shows the detailed retention rates of each AA specialty service after the MMRCI implementation.

**Table 2:** Retention by Specialty Service After MMCRI Implementation. *CC*, community care; *AA*, Ann Arbor

	Specialty service	Total consults	% Retained by AA
Battle Creek			
	Otolaryngology - CC	860	-
	Otolaryngology - AA	632	42%
	General surgery - CC	813	-
	General surgery - AA	248	23%
	Hematology - CC	297	-
	Hematology - AA	411	58%
	Nephrology - CC	1058	-
	Nephrology - AA	371	26%
	Neurosurgery - CC	781	-
	Neurosurgery - AA	395	34%
	Oncology - CC	824	-
	Oncology - AA	66	7%
	Pulmonary - CC	548	-
	Pulmonary - AA	197	26%
	Radiation oncology - CC	78	-
	Radiation oncology - AA	6	7%
	Rheumatology - CC	258	-
	Rheumatology - AA	191	43%
	Vascular surgery - CC	625	-
	Vascular surgery - AA	266	30%
	**Total consults to CC**	**6142**	-
	**Total consults to AA**	**2783**	**31.2%**
Saginaw			
	Otolaryngology - CC	445	-
	Otolaryngology - AA	299	40%
	Hematology - CC	452	-
	Hematology - AA	292	39%
	Nephrology - CC	447	-
	Nephrology - AA	238	35%
	Neurosurgery - CC	79	-
	Neurosurgery - AA	30	28%
	Radiation oncology - CC	70	-
	Radiation oncology - AA	4	5%
	Rheumatology - CC	36	-
	Rheumatology - AA	23	39%
	Vascular surgery - CC	330	-
	Vascular surgery - AA	131	28%
	**Total consults to CC**	**1859**	-
	**Total consults to AA**	**1017**	**35%**

**Figure 2:**
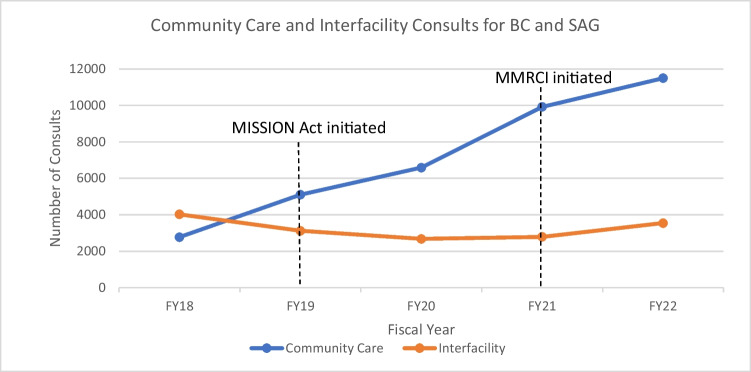
Community Care and Interfacility Consults to Ann Arbor.

Figure 2 illustrates the combined trend in CC and IFC volumes from both SAG and BC. After the implementation of the MISSION Act in FY 2019, there was a sharp increase in CC referrals. With the introduction of the MMRCI in FY 2021, there was an increase in IFCs to AA FY 2022 with a resultant decrease in the year-to-year acceleration of CC referrals.

An estimation of dollars redirected to the VA system due to retention of referrals is shown in Table 3, tabulated by specialty service and site. BC retained an estimated $17,574,970 with SAG retaining an estimated $6,530,281 for an overall cost avoidance of $24,105,251. The highest cost avoidance at both sites was observed in Hematology and Otolaryngology due to the combination of high consult volumes and SEOC cost, particularly for Hematology.

**Table 3:** Estimated VA Dollars Retained Based on Average SEOC Cost

Site	Specialty service	Total consults retained	Avg SEOC cost (USD)	Estimated cost avoidance (USD)	
Battle Creek	Otolaryngology	632	$4110	$2,597,520	
	General Surgery	248	$3874	$960,752	
	Hematology	411	$17,930	$7,369,230	
	Nephrology	371	$2420	$897,820	
	Neurosurgery	395	$6472	$2,556,440	
	Oncology	66	$9950	$656,700	
	Vascular Surgery	266	$4678	$1,244,348	
	Pulmonary	197	$4770	$939,690	
	Radiation oncology	6	$12,601	$75,606	
	Rheumatology	191	$1786	$341,126	
				**BC site total**	**$17,574,970**
Saginaw	Otolaryngology	299	$4106	$1,227,694	
	Hematology	292	$14,165	$4,136,180	
	Nephrology	238	$1419	$337,722	
	Neurosurgery	30	$7317	$219,510	
	Radiation oncology	4	$11,574	$46,296	
	Rheumatology	23	$1781	$40,963	
	Vascular surgery	131	$4741	$621,071	
				**SAG site total**	**$6,530,281**
				**Grand total**	**$24,105,251**

As shown in Table 4, sampling of AA specialty clinic and referring site community new patient wait times occurred on 8/28/2022 when the pilot project was thought to be in a steady state. Despite the increased influx of consults throughout the initiative, AA clinic wait times were superior to their community counterparts in every category except Nephrology, Neurosurgery, and Vascular Surgery. Four AA clinics offered wait times that were 50% or less of community options (Otolaryngology, Hematology, Radiation Oncology, Rheumatology). As expected, BC community wait times were universally superior to those from SAG due to SAG’s rural location.

**Table 4:** VA and Community Specialty Clinic New Patient Wait Times After MMRCI Implementation. *BC*, Battle Creek; *SAG*, Saginaw; *CC*, community care; *AA*, Ann Arbor

Specialty service	Average new patient wait time (days)^*^		
	AA VA clinic	BC comm clinic	SAG comm clinic
Otolaryngology	31.5	70.4	69.1
General surgery	25.2	46.3	33.7
Hematology	40.4	79.6	73.5
Nephrology	95.9	54.1	101.8
Neurosurgery	73.9	59.4	72.7
Oncology	35.3	40.6	71.1
Pulmonary	40.7	66.8	99
Radiation oncology	13	30	49.2
Rheumatology	62.7	144.7	136.6
Vascular surgery	53.5	48.4	63.8

As wait times were a secondary balancing measure, they were not collected prior to our intervention, and thus are currently unavailable for comparison.

## DISCUSSION

Access to care has been a central reason veterans choose care outside of the VA despite evidence that VA care is equivalent or superior in safety and effectiveness versus non-VA settings.^11,12^ While the 2014 VA Choice Program and the MISSION Act of 2018 endeavored to improve access to care, neither program was universally successful in this regard, and since 2014 access to VA care has continued to improve.^8,13^ Even with this gainful dynamic, the lack of a coordinated, standardized IFC referral process has stymied veteran retention within the VA. MMRCI has successfully overcome many of the clinical and administrative barriers, streamlining care coordination among VA facilities and connecting with veterans themselves.

MMRCI has several strengths, most notably its referral process efficiency. Central coordination of referral triage allows for standardization among sites and alleviates administrative burden for referring PCPs. While MMRCI does add an extra step in IFC processing, with a dedicated team to rapidly triage veteran preferences and the ability to directly order needed tests in sending site CPRS systems, the referrals received downstream by AA specialty clinics (or CCP) are more complete.

Second, early veteran engagement has ensured awareness of all care options both within and outside the VA, allowing for informed decision-making. Not only does this veteran-centered approach result in retaining dollars within the VA medical system, but it also reduces the risk of healthcare-related financial toxicity to the veteran. Furthermore, facilitating care within a single healthcare system allows for comprehensive case management, avoiding fracturing of care and poor communication among healthcare providers which are common sources of medical errors and delays.^14,15^ While retention rates of greater than 32% solely by making veterans aware of local VA care options are impactful, we believe that with further evaluation of barriers and targeted support (such as transportation and lodging), retention rates of 50% or more are possible.

Another strength of the MMRCI is the large fiscal impact it can have at the VA, as evidenced by the estimated CC cost avoidance of over $24 million dollars in just its first 14 months. While we acknowledge our estimated cost avoidance calculations do not represent a direct transition of funds from CC to VA facility operating budgets, they do signify a substantial amount of workload that will be distributed to the facility in future years’ budgets via the Veteran Equitable Resource Allocation model. For those health centers in rural areas, the potential budgetary savings for a program like MMRCI is considerable, as a disproportionate amount of their budgets is spent on CC. In 2021, BC spent 32.6% of its budget on CC, which was second only to staff salaries.^16^ We note that while our cost avoidance projection does not factor in the cost of delivering services to the veterans who opted to stay within the VA, many of the VA’s operational costs are fixed and no incremental clinical or MMRCI staff were hired to maintain this effort during our pilot period. However, we do expect that over time investments in MMRCI and specialty care clinics will be required to ensure VA capacity expands with demand.

The VA Michigan Market is geographically large, but the MMRCI has taken advantage of virtual options to deliver care despite these distances. In-home video visits are routinely available and allow for caregivers to also be involved in the veteran’s care without traveling. In our experience, leveraging a “virtual first” approach can allow veterans to be seen in specialty clinics efficiently with minimal initial commitment. Upon developing a rapport with their providers, the veterans experience better engagement and utilization of VA resources if face-to-face services are subsequently required. Although many rural veterans are hampered by a lack of access to broadband internet,^17^ clinical video telehealth offerings at many VA clinics ensure a stable video connection while minimizing travel.

The VA Michigan Market has a long history of collaboration which undoubtedly contributed to the success of the MMRCI. While the state of Michigan is currently in VISN 10, previously AA, SAG, and BC belonged to VISN 11; thus, the referral patterns involving these health systems were already in place. Without this level of interfacility rapport, it may be difficult for other VA health systems to replicate the MMRCI process. The Detroit VA has recently joined the MMRCI and represents a 4th Michigan Market health system in this effort. We anticipate that medical and surgical specialty service lines will continue to be added until all are participating in the MMRCI.

We acknowledge that our MMRCI model has limitations. Firstly, given the collaboration history described above, veterans with MMRCI consults may already be receiving care in AA, and thus, our initial retention rates may be higher than experienced for VA sites implementing similar programs de novo. Secondly, our evaluation of MMRCI lacks perspectives of the veterans’ and the VA PCPs’ experiences with the program. These are both valuable components for success moving forward, and we are actively working on obtaining satisfaction data. Thirdly, the cost avoidance analysis utilizing the average SEOC cost is only an estimate of the maximum potential cost avoidance for a facility and not actual care delivered. As SEOC reimbursements can be highly variable, we intend to transition to utilizing a median SEOC cost, along with direct VA workload calculations for the care delivered.

Lastly, our new patient clinic wait time comparison was only evaluated at a single point in time, rather than as a real-time dynamic metric, and we were unable to obtain historical wait time data to do a pre- and post-intervention analysis. However, clinic wait time comparisons were not the primary focus of our intervention but instead intended to serve as a measure of AA clinic capacity to guide further clinic resource investment.

In conclusion, the MMRCI is an example of an enhanced, well-coordinated, veteran-centered interfacility collaboration which has led to the successful retention of veterans within the VA system. Focusing on central care coordination and veteran engagement in the referral process are keys to the success of the MMRCI, along with leveraging existing referral patterns between nearby VA facilities. We would highly encourage other VA sites to explore the creation of similar programs in concert with their local VISN partners in order to provide all veterans with a robust VA option for care.
